# Maternal and fetal effects of COVID-19 virus on a complicated triplet pregnancy: a case report

**DOI:** 10.1186/s13256-020-02643-y

**Published:** 2021-02-18

**Authors:** Maryam Rabiei, Tahereh Soori, Amene Abiri, Zohreh Farsi, Arshia Shizarpour, Reihaneh Pirjani

**Affiliations:** 1 Arash Hospital, Tehran University of Medical Sciences, 1416753955 Tehran, Iran; 2 Medical School, Tehran University of Medical Sciences, Tehran, Iran

**Keywords:** Case report, COVID-19 virus, Placental insufficiency, Triplet pregnancy

## Abstract

**Background:**

Coronavirus disease 2019 (COVID-19), the global pandemic that has spread throughout the world, is caused by severe acute respiratory syndrome coronavirus 2 (SARS-CoV-2). Given the limited scientific evidence on the manifestations and potential impact of this virus on pregnancy, we decided to report this case.

**Case presentation:**

The patient was a 38 year-old Iranian woman with a triplet pregnancy and a history of primary infertility, as well as hypothyroidism and gestational diabetes. She was hospitalized at 29 weeks and 2 days gestational age due to elevated liver enzymes, and finally, based on a probable diagnosis of gestational cholestasis, she was treated with ursodeoxycholic acid. On the first day of hospitalization, sonography was performed, which showed that biophysical scores and amniotic fluid were normal in all three fetuses, with normal Doppler findings in two fetuses and increased umbilical artery resistance (pulsatility index [PI] > 95%) in one fetus. On day 4 of hospitalization, she developed fever, cough and myalgia, and her COVID-19 test was positive. Despite mild maternal symptoms, exacerbated placental insufficiency occurred in two of the fetuses leading to the rapid development of absent umbilical artery end-diastolic flow. Finally, 6 days later, the patient underwent cesarean section due to rapid exacerbation of placental insufficiency and declining biophysical score in two of the fetuses. Nasopharyngeal swab COVID-19 tests were negative for the first and third babies and positive for the second baby. The first and third babies died 3 and 13 days after birth, respectively, due to collapsed white lung and sepsis. The second baby was discharged in good general condition. The mother was discharged 3 days after cesarean section. She had no fever at the time of discharge and was also in good general condition.

**Conclusions:**

This was a complicated triplet pregnancy, in which, after maternal infection with COVID-19, despite mild maternal symptoms, exacerbated placental insufficiency occurred in two of the fetuses, and the third fetus had a positive COVID-19 test after birth. Therefore, in cases of pregnancy with COVID-19 infection, in addition to managing the mother, it seems that physicians would be wise to also give special attention to the possibility of acute placental insufficiency and subsequent fetal hypoxia, and also the probability of vertical transmission.

## Introduction

Coronavirus disease 2019 (COVID-19), caused by the severe acute respiratory syndrome coronavirus 2 (SARS-CoV-2), is a global pandemic that has spread throughout the world. Unfortunately, there is still limited scientific evidence on the manifestations and potential impact of this virus on pregnancy. In this article, we aim to report the maternal and fetal effects of the virus on a complicated triplet pregnancy.

## Case

A 38 year-old Iranian woman with a triplet (three chorionic and three amniotic) pregnancy was hospitalized at 29 weeks and 2 days gestational age due to one-time high blood pressure at 140/90mmHg and elevated liver enzymes. A week before admission, she had been hospitalized in another hospital for about 5 days to evaluate the cause of increased liver enzymes. She had a 2-year history of primary infertility and had become pregnant by ovulation induction, and also had a 5-year history of hypothyroidism, which was euthyroid during pregnancy. She had also been diagnosed with gestational diabetes a month before admission, which was being treated with 16 units of insulin daily (6 units Levemir and 10 units NovoRapid), so her blood glucose levels were maintained in a normal range, and glycated hemoglobin concentration was 5.6 %. She was at risk for preeclampsia due to her advanced age and triplet pregnancy, so aspirin was administered. On the other hand, due to hospitalization for more than 3 days, she was at increased risk of deep vein thrombosis and was treated with enoxaparin. Additionally, given the possibility of iatrogenic preterm delivery, she had received a course of betamethasone for fetal lung maturation.

Two weeks before hospitalization, liver enzymes increased to four times the normal levels: alanine amino transferase (ALT) 218 units per liter (U/L) and aspartate amino transferase (AST) 283 U/L. After a thorough evaluation and ruling out preeclampsia, and due to a probable diagnosis of gestational cholestasis, she was treated with ursodeoxycholic acid (300 mg twice a day) from 1 week before hospitalization. Lab tests at admission included the following: ALT = 94 U/L, AST = 57 U/L, total bilirubin = 0.7, direct bilirubin = 0.1, and lactate dehydrogenase (LDH) = 276 U/L. Other tests including white blood cell count, hemoglobin, platelet, serum creatinine, and urinalysis were in the normal range. She underwent 24-hour Holter blood pressure monitoring, and among all measurements, only 18.8% of systolic and 15.6% of diastolic blood pressure exceeded the set limit of 140 and 90 mmHg, respectively. Echocardiographic findings were quite normal, and 24-hour urine protein was also reported in the normal range. On the second day of hospitalization, an ultrasound exam was performed, which showed that biophysical scores and amniotic fluid were normal in all three fetuses. One of the fetuses had increased umbilical artery resistance (pulsatility index [PI] > 95%) and estimated weight below 5%, but umbilical cord and middle cerebral artery findings were normal in the other two fetuses. On the fourth day of hospitalization, the patient developed fever and cough. The following day, due to the persistent fever and cough and also an onset of myalgia, real-time reverse transcriptase polymerase chain reaction (RT-PCR) was conducted on nasopharyngeal swabs for SARS-CoV-2 nucleic acid. All steps including sample collection, processing and laboratory testing were based on World Health Organization (WHO) guidelines. Chest X-ray and computed tomography scan were not performed due to the patient’s lack of consent.

**Fig. 1:**
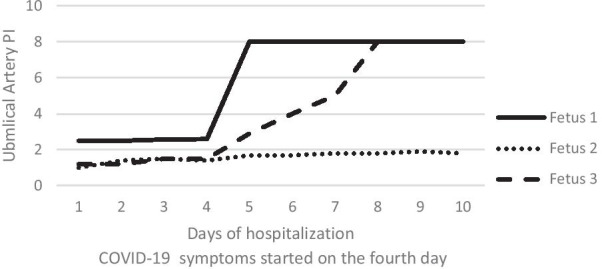
Fetal umbilical artery resistance during hospitalization

The result of RT-PCR was positive for the SARS-CoV-2 virus. However, the patient’s clinical symptoms were mild. She had a mild fever, with maximum temperature of 38.3 °C. She had no complaint of shortness of breath, diarrhea, nausea or vomiting, her respiratory rate was 18–20 per minute, and oxygen saturation was above 95% at all times. On the same day that she developed clinical symptoms, an ultrasound exam was performed: the fetus who already had increased umbilical artery resistance showed an exacerbated condition involving absent umbilical artery end-diastolic flow, and another fetus showed umbilical artery resistance (PI > 95%), but umbilical cord and middle cerebral artery findings were normal in the third fetus, and biophysical scores and amniotic fluid were normal in all three fetuses. Based on these findings, the patient underwent serial ultrasound exams, and unfortunately, exacerbated umbilical flow resistance in the second fetus also progressed to absent umbilical artery end-diastolic flow (Fig. 1). However, Doppler and biophysical scores were normal in all fetuses. Finally, 6 days after the beginning of clinical symptoms, the biophysical scores declined in the two fetuses with absent umbilical artery end-diastolic flow, so the patient underwent cesarean section due to rapid deterioration of fetal conditions and exacerbated placental insufficiency. The first baby was born weighing 1320 g with umbilical cord pH 7.25, and her 5-minute APGAR [appearance, pulse, grimace, activity, respiration] score was 4; the second baby was born weighing 1600 g with umbilical cord pH 7.23, and his 5-minute APGAR score was 7; the third baby was born weighing 1250 g with umbilical cord pH 7.21, and her 5-minute APGAR score was 6. In order to protect the babies from infection with the virus, delayed cord clamping was not performed, skin-to-skin contact of mother and babies was not practiced, and the babies were separated from the mother immediately after birth. All three babies were intubated and were admitted to the neonatal intensive care unit (NICU), where they were kept in separate, isolated rooms. RT-PCR of nasopharyngeal swabs for SARS-CoV-2 nucleic acid was carried out for all three newborns immediately after birth. The mother was discharged 3 days after cesarean section, and 2 weeks later she had recovered completely without any complications.

Two of the babies, weighing 1250 and 1320 g, each received three doses and the other baby received two doses of surfactant. All three newborns developed clinical symptoms of sepsis and pulmonary hemorrhage. The primary results of the COVID-19 test were negative for all three newborns. Because of the poor general conditions of the newborns, and considering the false-negative probability of the initial test, the COVID-19 test was repeated immediately after receiving the first test results, and the result was positive for the baby who weighed 1600 g and also had better umbilical cord and placental circulation before birth. Unfortunately, we did not examine the umbilical cord blood and amniotic fluid samples for the virus, so we cannot conclusively link the COVID-19 RT-PCR positive test in this fetus to vertical transmission, but because the babies were completely isolated and had no suspected exposure during the period between the two tests, the possibility of vertical transmission cannot be ignored.

The baby who weighed 1320 g died 3 days after birth with collapsed white lung and sepsis. The baby who weighed 1250 g also had symptoms of sepsis and died 13 days after birth. The baby who weighed 1600 g and had a positive COVID-19 test eventually recovered and was discharged in good general condition 3 weeks after birth.

## Discussion

We report the case of a woman with a triplet pregnancy who was infected with COVID-19. Despite the presence of some comorbidity, she had only mild symptoms of COVID-19 infection, but rapid and progressive placental insufficiency occurred simultaneously with the peak maternal infectious disease symptoms. A COVID-19 test was positive in one baby, who was discharged in good condition 3 weeks after birth, and negative in two other babies, who died 3 and 13 days after birth.

A recently published study identified maternal age and underlying diseases as risk factors for severity of COVID-19 symptoms [1]; however, other studies have reported maternal mortality in women without underlying disease [2, 3]. In our previous prospective cohort study of COVID-19-infected pregnant women, we did not have any maternal deaths, and there were no differences in underlying disease between COVID-19-infected and non-infected pregnant women [4]. However, studies in this area are insufficient, and further research is needed.

Available data about COVID-19 vertical transmission is still contradictory. At the beginning of the pandemic, no vertical transmission was reported [5]; however, some cases of probable vertical transmission have recently been noted [6–8]. In a recent review of 50 studies, the virus test was positive in 17 cases of neonatal secretions, eight cases of placental tissue, three cases of breast milk and one case of amniotic fluid, and anti-SARS-CoV-2 antibodies were positive in three infants [9]. Therefore, the possibility of vertical transmission should be considered.

Delayed positive tests in newborns have also been reported in other studies [2, 6–8]. In most reports, the positive test occurred with a delay of 16–72 hours after birth; therefore, we recommend that all infants born to COVID-19-infected mothers be retested within the next hours if the test immediately after birth is negative. In our study, SARS-CoV-2 PCR tests for the babies were negative immediately after birth, and interestingly, changed to delayed positive in one baby who during the time between the first and second tests was completely isolated and had no suspected exposure. Unfortunately, we did not test the placenta, umbilical cord or amniotic fluid samples for the virus, and this is a limitation of our study.

Our case was a triplet pregnancy. Most previous studies are in singleton pregnancies, although there are also some reports of twin pregnancies [10–12]. 

Some have hypothesized that maternal respiratory failure and hypoxia may transiently reduce uterine placental blood flow [13, 14], but in our case, the mother did not have severe illness. However, there was severe placental insufficiency in two of the fetuses, both of whom had negative COVID-19 test results. Interestingly, it was the largest fetus with better placental circulation who was infected. On the other hand, although this woman had several underlying factors for placental insufficiency, and umbilical artery resistance was noted in one of the fetuses before maternal infection, the rapid and progressive placental insufficiency occurring during the last days of pregnancy and simultaneously with the peak maternal infectious disease should be taken into account. Although this pregnancy was a complicated one and there were several risk factors for placental insufficiency, the conceivable and hypothetical impact of COVID-19 on uterine circulation and fetal hypoxia should also be considered.

## Conclusion

There are still insufficient data about COVID-19 in pregnancy. Our case was a complicated triplet pregnancy; however, COVID-19 symptoms in the mother were mild. Given the rapid and progressive placental insufficiency after COVID-19 infection, it seems prudent that in cases of pregnancy with COVID-19 infection, in addition to assessing and managing the mother, special attention should be given to the possibility of acute placental insufficiency and subsequent fetal hypoxia. The possibility of vertical transmission should also be considered.

## Data Availability

Data are available from the electronic health record at Arash hospital.
